# RNA, the epicenter of genetic information: wirtten by John Mattick and Paulo Amaral, CRC Press, Boca Raton, 2022

**DOI:** 10.1080/15476286.2022.2108246

**Published:** 2022-08-05

**Authors:** Witold Filipowicz

**Affiliations:** Friedrich Miescher Institute for Biomedical Research, Basel, Switzerland

Many surprising discoveries in the last few decades have radically changed our view of gene expression and its regulation, particularly concerning the role of RNA. Starting in the mid-1970s, we have witnessed the discovery of mosaic genes and splicing, the unearthing of RNA-mediated catalysis, the characterization of different classes of non-coding regulatory RNAs, and a plethora of RNA modifications and RNA-editing reactions. In parallel, the development of novel sequencing technologies has led to the sequence of the human genome and the genomes of many other organisms. It has also resulted in a more profound account of transcriptomes, expressed not only in different organs or at different developmental stages but also in individual cells. Together, such technological breakthroughs have provided many new insights into the organization, dynamics, and expression of complex genomes, and have also revealed the basis of organismal development and cell differentiation.

The forthcoming book ‘RNA, the Epicenter of Genetic Information’ by John Mattick and Paulo Amaral (https://www.routledge.com/RNA-the-Epicenter-of-Genetic-Information/Mattick-Amaral/p/book/9780367567781) is much more than what its title might suggest. It is not only a description of our current understanding of the role of RNA in cell and developmental biology but is also a useful history of molecular biology. The authors chronicle the major developments that established DNA as a hereditary material, that determined the principles of cellular protein synthesis (with ribosomes and transfer and messenger RNAs (!) involved), and that led to deciphering of the genetic code. They follow this with an account of how RNA gradually – despite the previous dominant protein-centric views – became recognized as the ‘Epicenter of Genetic Information’, where the transcription of non-protein-coding DNA into tens of thousands of regulatory RNAs plays a major role in the biology of complex organisms, their evolution, development, and function.

The book is truly monumental. I spent much time reading each chapter, mainly because I was continually confronted with new information or with references to papers about which I had no idea. I repeatedly ran to the library to update myself on the topic. This is not at all surprising given that the text includes six thousand references and hundreds of footnotes. It is remarkable how much the field of gene expression and RNA biology has grown in the last few decades. I have been working in this area since the mid-1960s, when I could familiarize myself with the entire field and essentially understand (almost) everything by simply reading Jim Watson’s 1965 textbook *Molecular Biology of the Gene*. It is stunning how the authors here have collated a huge amount of information into a logical sequence of events that depicts the evolution of our understanding of the organization and function of complex genomes, with RNA emerging more and more into the limelight.

Each of the eighteen chapters is a brilliantly written semi-autonomous essay on a particular segment of the RNA odyssey. The poetic titles of several chapters, such as *Halcyon Days, Worlds Apart, The Age of Aquarius*, and *All that Junk*, reflect the authors’ very personal connection to the history (and also future prospects) of RNA research. Their emotions are further shown in the way they deal with the early reluctance to accept new – often totally unexpected – findings that supported a central role of RNA in biology. I find it appropriate that the most important discoveries and technical breakthroughs are set into an historical context, where the main actors behind the findings are introduced together with the accompanying disputes. This is particularly relevant for those at the beginning of their careers who are overburdened with day-to-day duties at the bench. One can draw important lessons from accounts of how other scientists – many of them ‘Science Giants’ – have behaved.

John Mattick pioneered the concept that most of the genome in complex organisms, including introns, ‘intergenic’ regions and all sorts of repetitive elements (all considered by many to be just ”junk”) is dedicated to the production of regulatory RNAs with roles in epigenetic regulation, differentiation, and development. Many of Mattick’s predictions have been vindicated by discoveries of intron-encoded small nucleolar (sno) RNAs and micro (mi) RNAs, of the massive occurrence of RNA alternative splicing and polyadenylation, of the frequent transcription of both sense and anti-sense DNA strands, and – finally – of the existence of tens of thousands of long non-coding (lnc) RNAs, including enhancer RNAs (eRNAs) and transcripts from repetitive regions or pseudo-genes. I have frequently argued that John Mattick should be on the payroll of the *RNA Society*, acting as its spokesman by advertising the centrality of RNA.

Many pages of the book are devoted to controversies and paradoxes that have emerged as a corollary of new findings: the post-splicing-discovery ‘introns-early versus introns-late’ debates; the ‘junk or not junk’ disputes about non-protein-coding DNA that largely exceeds the coding DNA in complex organisms, and finally – when pervasive transcription of complex genomes was already evident – the question ‘do thousands of new lncRNAs reflect transcriptional noise or rather represent a new class of RNA regulators?’ These and other disputes are illustrated with interesting quotes from the adversaries that add much flesh to the discussion.

On some occasions, Mattick and Amaral seem to be rather annoyed by conservative views and the initial lack of acceptance of emerging new ideas. I think this is not necessary. Controversies and heated disputes are generally good for science as they drive its progress. By nature, I am also a rather conservative and sceptical person and I would argue that some concepts discussed in the book, not necessarily derived from the authors’ research, need caution and certainly more experimentation. For example, although evidence exists that independent RNAs originating from 3’-untranslated regions (UTRs) of vertebrate mRNAs can exert biological effects in *trans* when artificially expressed, is not clear whether they do so – as the 3’-UTR *fragments* – in more physiological settings. Likewise, I would welcome more evidence and also mechanistic insights that support a nuclear role of miRNAs or the involvement of exosome-contained regulatory RNAs in information transfer between distant cells or tissues in vertebrates.

As already discussed, this epic book by Mattick and Amaral superbly reflects the continuing excitement about RNA research. RNA is a molecule with many faces. It can carry genetic information, act as a catalyst, be a target of allosteric regulation (riboswitches, responding to different metabolites, as an example), function as a scaffold in the assembly of ribonucleoproteins of different complexity, or guide itself or a cargo to a destination by base-pairing. This is not a complete list of RNA ‘capabilities’ by far and for more information you should read the book. I particularly recommend the last five chapters that discuss: the epigenome (Chapter 14), which also includes – as does Chapter 16 – an interesting section on enhancers and eRNAs; the programming of development (Chapter 15); the unusual complexity, and often uniqueness, of different RNA reactions in the brain (Chapter 17 ‘Plasticity’). Chapter 17 also contains an interesting discussion of transgenerational epigenetic inheritance, paramutations, and related phenomena, which generate a lot of interest despite being mechanistically ill-defined. Chapter 16 (‘RNA Rules') focuses to a large extent on the importance of RNA as a guiding molecule. The authors consider this as the key to exploiting the potential of thousands of regulatory RNAs to precisely direct generic effector proteins (e.g. chromatin modifying and remodelling enzymes or transcription factors) to related destinations, either chromatin components or even other RNAs. Given the huge developmental complexity of organisms and the even more sophisticated development and functioning of the human brain, the vast arrays and hierarchies of regulatory RNAs, combined with epigenetic control of gene expression, may reveal the source of the information that controls these tremendously complicated processes? The chapters listed above, as well as the concluding Chapter 18 (‘Beyond Jungle of Dogmas’), are more philosophical and understandably also more speculative than others. But they provide much food for thought about ‘dark matter’ areas in our understanding of evolution and the functioning of complex genomes.

I would have liked to find a chapter in the book devoted to the continuously ‘expanding world’ of RNA-binding proteins (RPBs). Some are briefly discussed in Chapter 16 in connection with chromatin regulation by RNA, with their role in the formation of phase-separated domains (PSDs: also known as Liquid-Liquid Phase Separation, LLPS), and with neurodegenerative pathologies associated with expansion of short intragenic repeats. It would be beneficial to have more information about the evolution and function of RBPs, and perhaps also about RNA helicases/ATPases, as both protein families intimately co-operate with RNA. Did the increase in the complexity of the two protein families occur in parallel to a gradual increase in the complexity of RNAs expressed from eukaryotic genomes? Did any new paradigms of RNA recognition or RBD and helicase gene organization appear at certain points in evolution?

The publication of ‘RNA, the Epicenter of Genetic Information’ is very timely. We are witnessing a true, fully justified explosion of research on RNA. In principle, we have to understand the function of a new genome that encodes all sorts of regulatory RNAs. After decades spent on dissecting the protein-coding genes and the completion of the human genome project about 20 years ago, we still do not know the function of many human genes. Despite enormous technological progress, establishing specific functions of genes encoding regulatory RNAs will not be easy. These genes have no open reading frames to interrupt or AUG initiation codons to mutate. Moreover, the common occurrence of genes nested within other genes, of overlapping genes, and of frequent situations when RNA is expressed from both complementary strands of DNA make studies of the activity of any gene quite difficult. But do not panic. RNA afficionados like John Mattick and Paulo Amaral, joined by hundreds of others, will find a way to proceed. I wholeheartedly recommend this book to anybody interested in the biology of RNA, in evolution, and in the organization and function of complex genomes. As correctly stated in the publisher’s announcement, this book provides a thought-provoking retrospective and forward-looking perspective for advanced students and professional researchers in a broad area of biology.

